# Molecular Genetics of Alcohol Dependence and Related Endophenotypes

**DOI:** 10.2174/138920208786241252

**Published:** 2008-11

**Authors:** Yann Le Strat, Nicolas Ramoz, Gunter Schumann, Philip Gorwood

**Affiliations:** 1 INSERM U675, IFR02, Université Paris 7, 16 Rue Henri Huchard, 75018 Paris, France; 2 Section of Addiction Biology, Institute of Psychiatry, Kings College, De Crespigny Park, London SE5 8AZ, England; 3 AP-HP, Service de Psychiatrie, Hôpital Louis Mourier, 178 Rue des Renouillers, 92700 Colombes, France

**Keywords:** Alcohol dependence, association, dopamine, endophenotype, GABA, linkage, proteomics, transcriptomics.

## Abstract

Alcohol dependence is a worldwide public health problem, and involves both environmental and genetic vulnerability factors. The heritability of alcohol dependence is rather high, ranging between 50% and 60%, although alcohol dependence is a polygenic, complex disorder.

Genome-wide scans on large cohorts of multiplex families, including the collaborative study on genetics of alcoholism (COGA), emphasized the role of many chromosome regions and some candidate genes. The genes encoding the alcohol-metabolizing enzymes, or those involved in brain reward pathways, have been involved. Since dopamine is the main neurotransmitter in the reward circuit, genes involved in the dopaminergic pathway represent candidates of interest. Furthermore, gamma-amino-butyric acid (GABA) neurotransmitter mediates the acute actions of alcohol and is involved in withdrawal symptomatology. Numerous studies showed an association between variants within GABA receptors genes and the risk of alcohol dependence.

In accordance with the complexity of the “alcohol dependence” phenotype, another field of research, related to the concept of endophenotypes, received more recent attention. The role of vulnerability genes in alcohol dependence is therefore re-assessed focusing on different phenotypes and endophenotypes. The latter include brain oscillations, EEG alpha and beta variants and alpha power, and amplitude of P300 amplitude elicited from a visual oddball task.

Recent enhancement on global characterizations of the genome by high-throughput approach for genotyping of polymorphisms and studies of transcriptomics and proteomics in alcohol dependence is also reviewed.

## INTRODUCTION

Alcohol dependence is a complex addictive disorder, affecting 5.4% of the general population during lifetime [1]. Alcohol dependence (Online Mendelian Inheritance in Man #103780) represents a complex and heterogeneous phenotype, with behavioural, as well as psychological, pharmacological, and medical components. Aggregation and adoption researches support the role of a genetic component in alcohol dependence. Twin studies estimate that the additive heritability ranges between 50% and 60% [2]. However, the exact transmission mode remains unknown, but more than one gene is likely to be involved (i.e. pauci- or poly-genic disorder).

This paper focuses on current studies on the genetics of alcohol dependence. After an overview of genetic approaches, including genome-wide scans and candidate genes analyses, we will review post gene methods, such as expression profile studies of genes differentially expressed in phenotypes relevant for alcohol dependence.We will particularly discuss the results from two emerging fields, namely genomic and proteomic approaches of alcohol dependence. 

## GENETICS OF ALCOHOL DEPENDENCE

The identification of genes involved in alcohol initiation, use, abuse and dependence has been investigated by two complementary genetic methods, namely (i) genome wide scan analyses, with no a priori assumptions about which genes might be involved, and (ii) the genotyping of candidate genes of interest. About fourteen-hundreds papers came out from the PubMed/NCBI database with the key words “alcohol, dependence, gene”. This review is not an exhaustive view of linkage studies on alcohol dependence, neither a cover of all genes possibly affecting the risk for alcohol dependence. We have rather decided to focus on particularly relevant linkage and candidate genes studies. For a more complete review refers to Goldman *et al*. [3] and Ramoz *et al*. [4]. We also excluded gene environment interactions of our review, and refer the reader to Gorwood *et al*. [5].

### Genome-Wide Scans of Alcohol-Dependence

Recently, linkage studies have identified several chromosomal regions, supporting the involvement of loci on chromosomes 1, 2, 4, 5, 6, 7, 12, 14, 16 and 17 in alcohol dependence [3, 4, 6]. Most of these results were found on alcohol dependent families from the collaborative study on the genetics of alcoholism (COGA). The COGA consists of large pedigrees ascertained using probands who met diagnostic not only for alcohol dependence from the Diagnostic and Statistical Manual of Mental Disorders [7], but also for Research Diagnostic Criteria [8], constituting probands with very specific diagnostic. Multiplex families with a least three affected first-degree relatives, including the proband, were defined as the COGA high risk sample. Linkage peak on chromosome 4q and 7q31-35 are the most consistently replicated among COGA samples [9-12]. The 4q region contains several genes encoding subunits of the receptors for the gamma-amino butyric acid (GABA), the major inhibitory neurotransmitter [13]. GABA biological pathway is probably involved in alcohol dependence and tolerance, variants of GABRA gene being associated with alcohol dependence as discussed later on.

A non-synonymous coding single nucleotide polymorphism in the *hTAS2R16* gene, located on the 7q chromosomal region, shows significant association with alcohol dependence and with a related phenotype, the maximum number of drinks ever consumed in 24 hours in the COGA sample, especially in African Americans sub-sample [14]. The *hTAS2R16 *gene codes for a bitter receptor and this SNP is associated with a diminution of the sensitivity to bitter-taste stimuli. These findings are consistent with previous investigations of the relationship between taste perception and alcohol dependence. Indeed, in animal models, the knock-out of several genes involved in taste transduction is consistently associated with a reduction in alcohol intake [15]. Another candidate gene in the 7q locus is the *CHRM2* gene. It codes for a Cholinergic muscarinic 2 receptor and shows significant association not only with alcohol dependence and the maximum number of drinks ever consumed [16], but also with other related phenotype including personality traits [17], cognitive functions [18, 19] or electrophysiological endophenotypes [20].

Furthermore, in a subset of 1364 individuals (N=123 families), significant linkage was recently found for quantitative traits related to alcohol dependence, on two novel chromosome regions 2q and 10p [21], with a logarithm base 10 of odds score (i.e. LOD score) above 3.3. Such a LOD score assumes a type I error rate below 5% [22]. The two regions encompass several candidate genes, including the gene coding for the 5-HT_2B_ receptor located on 2q, which was previously associated with drug abuse [23], and the GAD2 gene on chromosome 10p region, which encodes the enzyme involved in the synthesis of GABA recently associated to alcohol dependence [24].

Studies upon genetically isolated population, such as Native American populations, are very promising due to the high homogeneity of the genetic background, and, in this case, a high prevalence of alcohol dependence [25]. Linkage analysis in Native Americans provided evidence for loci on chromosomes 5, 6, 12, 15 and 16 [3, 4, 26], and allowed the identification of GABA receptor genetic variants as a risk factor for alcohol dependence (see below) [27].

### Candidate Genes

#### Candidate Genes in the Metabolism of Alcohol

##### ADH Genes

The chromosome 4q region surrounding the alcohol dehydrogenase (ADH) gene was linked in a genome-wide scan with the maximum numbers of drinks ever consumed in 24 hours, a phenotype associated with alcohol dependence [28]. The role of ADH is to convert alcohol into acetaldehyde. The seven ADH genes cluster on chromosome 4q (from 5’ to 3’ region: *ADH7-ADH1C-ADH1B-ADH1A-ADH6-ADH4-ADH5*). However, only two exhibit functional polymorphisms, ADH1B*2 and ADH1C*2 alleles, and confer an increase in alcohol degradation rate [29, 30]. The class I enzymes (encoded by *ADH1A*, *ADH1B* and *ADH1C*) and the class II enzyme (encoded by *ADH4*) are significant contributors to ethanol metabolism, at least *in vitro* [30]. ADH1B*2 is relatively common among Asian population [31], as well as in Israeli Jews [32]. It is associated with a diminution in alcohol consumption and therefore has a protective effect against alcohol dependence [31]. ADH1C*2 has protective effects on liver cirrhosis and alcohol chronic pancreatitis [33], an is also associated with a lower rate in alcohol dependence among Asian population, maybe because *ADH1B* and *ADH1C* are in linkage disequilibrium[34]. Indeed, *ADH1B *and* ADH1C *are separated by a distance of only 15Kb in the cluster of *ADH* genes. Contrasting with their major effect *in vitro*, *ADH1C *and *ADH1B* genes have minor effects *in vivo*, suggesting a possible role of other genes of the ADH cluster. Indeed, the effect of the quantitative trait loci (QTL) linked to the ADH region gene account for 64% of the genetic variance in alcohol metabolism [35]. For example, *ADH4* contribute to 30% of the total ethanol oxidizing capacity of the liver [30]. Recent findings suggest evidences of association with alcoholism [30, 36], as well as with personality traits [37], although among non-coding SNPs. Similarly, a haplotype of the ADH7 gene contribute in the inter-individual variation in alcohol metabolism [38], and is associated with alcohol dependence [39], drug dependence [40] as well as with personality traits [41].

##### ALDH Genes

Acetaldehyde dehydrogenase (*ALDH*) genes encodes a protein that acts as a tetramer to oxidize acetaldehyde [3,4]. The blockage of ALDH with disulfiram leads to the accumulation of acetaldehyde in the blood, and results in a flushing syndrome, that is a reddening of the face, sudden sweating, vomiting and headache. This syndrome can also occur in subjects carriers of the ALDH2*2 variant, coding for an inactive enzyme. The deficient variant allele acts dominantly, thus ALDH2*1/ALDH2*2 heterozygous carriers have reduced enzyme activity, but the flushing reaction is more severe and immediate in ALDH2*2 homozygous carriers [42]. The ALDH2*2 is associated with a reduced rate of alcohol dependence in Asian population [43], and in both European and African Americans [44].

Interestingly, *ADH1B* and *ALDH2* genes have an additive effect on the risk of flushing, with no epistatic interaction involved, that is the protective effects conferred by one gene are independent of those conferred by the other [44].

#### Candidate Gene Involved in the Rewarding Circuits

Ethanol modulates several neurotransmitter systems involved in alcohol initiation, tolerance, preference, consumption, abuse and dependence [4]. These relationships interplay with neurocognitive functions, personality traits related either to alcohol seeking process or to subjective effects of ethanol. Regarding the neurobiological pathways involved in these processes, they are linked to the reward pathway, which mainly consisted of the dopaminergic projection of the ventral tegmental area on the nucleus accumbens. This dopaminergic pathway is modulated by several other neurotransmitters, including the GABA.

##### Dopamine Pathway

The ability of alcohol to increase brain dopamine concentration in the mesolimbic pathway has a key role in its reinforcing effects. Dopamine is also involved in withdrawal physiopathology, as suggested by the lower levels of striatal D2 receptors during alcohol withdrawal [45]. Several genes are involved in the neurobiological dopaminergic pathway, which have led researchers to assess different candidate gene polymorphisms. For instance, the single nucleotide variant TaqIA⁄ rs1800497 of the dopamine D2 receptor, *DRD2* gene, has been considered as a vulnerability gene for alcoholism in more than 40 studies, but with conflicting results, as suggested by meta-analysis [46, 47]. Interestingly, and in accordance with the role of dopamine in the detection of potential reward facets of external stimuli. the A1 allele carriers of the rs1800497 learned to avoid actions with negative consequences less efficiently [48]. Therefore, they are less likely to learn from the negative consequences of their alcohol consumption, and thus may be more likely to become dependent. This indirect involvement could partly explain the discrepant results observed in previous association studies. On the other hand, recent dense mapping of the DRD2 locus suggests association with adjacent genes, namely *ANKK1*, *TTC2* and *NRCAM1* [49, 50]. In fact, TaqIA variants change the amino acid in the ANKK1/X-kinase gene [51], and might confer functional biological consequences in alcohol dependence.

Recently, association between alcohol dependence and a SNP in the *DRD1* gene has been shown in a French population [52]. This is convergent with a previous association between DRD1 (among 5 genes) in a Korean population [53]. While there is evidence for the role of D3 receptor in alcohol intake in animal [54], genetic studies of dopamine receptor D3 and D4 genes failed to show associations with vulnerability to alcoholism [55-57].

The *DAT1* gene encodes the dopamine transporter (DAT) and represents a promising candidate gene in the dopaminergic system. This transporter is mainly located at the synapse of the presynaptic neuron and is responsible for dopamine reuptake. Human genetic studies report associations between variants of the *DAT1* gene and alcohol dependence with both positive [58, 59] and negative [60, 61] results. Indeed, the A9 variant of the *DAT1* is associated with more severe withdrawal symptomatology [62], and complications [63]. More specifically, this allele is associated with withdrawal seizures, probably through the modulation of neuronal excitability [64].

##### Gamma-Amino-Butyric Acid (GABA) Pathway

GABA is the major inhibitory neurotransmitter in the brain. The GABA receptor is a pentameric protein containing two α, two β, and one γ or δ subunit among 19 isoforms. This arrangement allows considerable diversity of the GABA receptor, but their observed number is in fact limited. Alcohol is an agonist of the GABA_A_ receptor, particularly receptors containing the α4 or α6 subunits in combination with β2 or 3 subunits and the δ subunit [65]. The GABA-ergic activity is reduced during alcohol withdrawal and could account for a part of the symptomatology. For example, in a mouse model, a mutant of the γ2 subunit is associated with an increased severity of ethanol withdrawal [66].

*GABRA6* variants were associated with alcohol dependence in different populations, including a Scottish [67], a German [68], a Finn and a Native American sample [66]. Variations in *GABRA2* were associated with alcohol dependence [69] as well as with illicit drug dependence [70] in the COGA sample, and this association was recently replicated in an independent sample [71].

## HUMAN POST-GENE STUDIES AND ANIMAL MODELS: SEARCH FOR ALCOHOL DEPENDENCE CANDIDATE GENES

Since almost a decade now, the traditional tools used in human genetic studies to identify alcohol dependence genes, genome-wide scans and candidate gene associations, have been helped by the high-throughput characterization of the expression patterns of the genes (transcriptome studies) and the proteins (proteome studies) from tissue samples. This tissue can be extracted from specific brain regions, of patients compared to controls, as well as from animal used as models of alcohol dependence, from the yeast to the murine models. Indeed, although there are several studies describing global gene expression changes associated with alcohol dependence, changes in the expression levels of proteins has not been characterized until recently. Characterization of the liver, brain and serum proteome is the next step toward the investigation of alcohol-dependent patients to discover new biological markers as well as prognostic tools.

### Transcriptome Studies

Whole-brain gene expression databases of five genetic mouse models with different alcohol consumption were examined [72]. A meta-analytic approach across data sets allowed the identification of 36 candidate genes for high and low amounts of alcohol consumption. Some of these genes were located within quantitative trait loci (QTLs) for human alcohol dependence susceptibility. Among them, 11 genes were clustered in only three gene families: GSTM (glutatione *S*-transferase activity), S100 class calcium-binding, and cytokine/proinflammatory activity (IL-1). Using microarray analyses of cerebellar tissue from mutant and wild-type mice exposed to alcohol, 109 genes were associated with either the development or the resistance to tolerance, or with the effects of chronic alcohol administration [73]. Specifically, six genes were both relevant to ethanol response and had modifications in micro array expression profiles further confirmed by quantitative real-time PCR. Of them, *Twik-1*, coding for a potassium channel, was associated with tolerance, and *JunB* and *Nur77*, members of an immediate early gene family and of the nuclear receptor family respectively, had an increased expression that persisted a long time after alcohol ingestion [73]. These two recent works illustrated the diversity of many gene expression studies on central nervous system from *in vitro* cell lines and *in vivo* animal models, especially in mice and rats [74-77]. It is important to note that transcriptome studies are usually coupled with phylogenetic and functional biological pathway studies for the analysed candidate genes, using GENE ONTOLOGY and INGENUITY packages, and may also integrate human genetic data in a more global approach called functional genomics, as illustrated in [74, 76, 77].

Due to the difficulty to have access to human post-mortem brain samples, the number of differential display and transcriptome studies on alcohol dependence, as well as the sample size of their studies, are modest. Nevertheless, several works have been published in the last years on independent samples of different brain regions, including temporal cortex, frontal cortex and nucleus accumbens of alcohol dependent patients and controls [78-82]. One of the first microarray gene expression studies identified a novel neuron-specific gene, *hNP22/TAGLN3*, over-expressed in the superior cortex of patients [78]. Significant differences in the expression levels were observed between cases and controls for genes involved in mitochondrial, ubiquitination and signalling pathways in the temporal cortex [79]. Genes encoding mitochondrial and metabolism proteins, as well as genes associated with neuroprotection and apoptosis, were also found in the prefrontal cortex of cases and not at the same level in controls [80]. Nucleus accumbens study revealed genes involved in the vesicle formation, in the structure, adhesion and migration of cells in the same patients [80]. Another expression analysis of other samples from the frontal cortex also showed dysregulation of genes implicated in metabolism, energy production, cell survival and communication [81]. This study was extended to additional cases and controls, and found altered expression of genes involved in myelination, ubiquitination, apoptosis, cell adhesion and neurogenesis [82].

The different pathways identified by the modifications of gene expression should help in the understanding of the molecular mechanisms of alcohol dependence, in particular by allowing the characterization of protein levels to reveal new biological markers, and therefore increase the chance to further understand the pathophysiology of this psychiatric disorder.

### Proteome Studies

Different methods in proteomics, such as liquid chromatography, mass spectrometry (MALDI-TOF and SELDI-TOF), and two-dimensional gel electrophoresis, have been recently carried out to identify and quantify proteins in the field of addiction. They have been used on cell lines, animal models, as well as on post-mortem tissues, in particular brain, of patients to characterize changes in protein expression in response to alcohol consumption, as presented in recent reviews [4, 83, 84]. Three recent works in this field could be emphasized, two on animal models and one in human post-mortem brain samples. In the first study, proteomic approach has been performed on liver from rats of inbred alcohol-preferring line (iP) exposed to alcohol or not [85]. The authors found 113 proteins with changes of expression levels. Seventy-one percent were enzymes and most of them are involved in metabolism pathways, including glycolysis, gluconeogenesis or fatty acid oxidation. These results, in particular the up-regulation of enzymes involved in antioxidant activity, are highly consistent with the known effects of alcohol on the liver. In the second study using mass spectrometry and confirmation by immunoblot analysis, levels of proteins in the serum of primates (cynomolgus monkey), trained to self-administrer ethanol, were compared between “drinking” and “abstinent” for periods of 6 months [86]. They observed a significant increase in apolipoproteins AI and AII. This observation is concordant with the detection of apolipoprotein in the serum of human ethanol consumption [87], and shows that this primate model represents a remarkable model to identify biological markers and maybe helpful to characterize the pathophysiology of alcohol dependence. In the third work, protein expression profiles from the white matter of the BA9 region were examined between alcohol dependant patients and controls [88]. Most of the differences were observed when comparing controls to severe alcoholic patients with complications (hepatic cirrhosis). Furthermore, some but not all spots of detected protein returned to control level in recovery alcohol dependent patients. This is of interest because these proteins could be involved in the regeneration of white matter after a prolonged abstinence. A total of 60 proteins were dysregulated in patients with alcohol dependence. Among these proteins, the level of hNP22 was significantly decreased (~3-fold) in complicated patients [88]. This biomarker was previously identified by its gene expression level in the superior cortex of patients [78] and corresponds to a hydrophilic, cytoplasmic protein involved in the cytoskeleton of the cell. Others proteins playing a role in the metabolism, mitochondria, energy production and ubiquitination were characterized [88], and were also previously identified by gene expression analysis.

Thus, a key area for proteomics consists in biomarker discovery that is the identification of protein expression which correlates with disease, treatment outcome or global prognosis, thus allowing patient-tailored therapy. In that view, the results presented above showed promising results. Furthermore, some biological markers were also found by gene profiling, and *vice versa*, which reinforce their probable implication in alcohol dependence. Finally, the last presented study point out the fact that the clinical features and phenotype of the patients need to be the most well documented to perform studies on comparable patients and homogenous groups. This lead to the novel strategy followed in genetic studies looking for susceptibility to psychiatric disorder which consists to identify specific traits that help to increase the homogeneity of the group of patients, when the disorder has a broad spectrum phenotype, such as alcohol dependence.

## ENDOPHENOTYPIC APPROACH

An endophenotype is a biological or a psychological characteristic transmitted with a disease, with a strong biological substrate and a higher heritability than the disease itself [89]. The required qualities of an endophenotype also include sensitivity and specificity, heritability, presence in un-affected relatives, state-independence, biological plausibility, sound psychometric properties and feasibility. The electrophysiological related features constitute the most studied endophenotype in alcohol dependence, and associates electroencephalography (EEG) variation and event-related potentials (ERPs).

### Brain Oscillations, EEG Alpha and Beta Variants and Alpha Power

EEG features are partially heritable and relatively stable among lifespan [90]. The alpha rhythm (7.5-12 Hz) is the posterior wave form, detected during alert relaxation. One common variant in EEG pattern concerns the alpha waves, since low voltage alpha (LVA) have previously shown a pattern of inheritance compatible with an autosomal dominant mode of inheritance [91]. LVA is associated with a familial history of alcohol dependence in subjects with no personal history of alcohol related problems. This finding was further confirmed on several ethnicities [92]. The beta waves (13–28 Hz) are also significantly different between alcohol dependent patients and controls. Moreover, the offspring of alcohol-dependent males also exhibited this variation [93], and was therefore used as an endophenotype in several genetic studies, in linkage analysis [94,95] and in candidate gene association [16, 20, 69].

### Amplitude of P300 Elicited from a Visual Oddball Task

The P300 amplitude reflects the allocation of attention, with the larger amplitude, the more attention allocated. It is measured during an oddball paradigm where patients are asked to detect target stimuli, and to ignore standard and non-target rare events. It is a complex wave, with at least two distinct components or ERPs, namely the P3a and the P3b waves. The P3a, the early component, is elicited by novel and unexpected stimuli, whereas the later P3b wave is elicited when context updating is necessary. 

The P300 is a highly heritable phenotype, since the estimated heritability ranges between 0.30 and 0.81 depending on the studies [96], and was therefore used as an endophentotype also in alcohol related disorders. Indeed, the P300 amplitude of the ERPs is reduced in the sons of alcoholic fathers, with no previous exposure to alcohol [97]. This variation was observed in several desinhibitory disorders, such as conduct disorder, attention-deficit hyperactivity disorder, oppositional defiant disorder, and antisocial personality disorder [98], suggesting that the P300 is associated with an intermediate phenotype, such as impulsivity, rather than with alcohol dependence itself.

## CONCLUSIONS

Our current knowledge on the neurobiological substrate of alcohol dependence leads to propose a high number of candidate genes. Association and linkage studies of the genes encoding proteins involved in alcohol metabolism (e.g. ADH, ALDH) and in the rewarding circuits, including dopamine (DRD2, DAT1) and GABA (GABRA2, GABRA6) neurotransmitter biological pathways, are the most replicated findings. It is important to note that novel candidate genes for alcohol dependence were identified from animal models. Thus, the drosophila *hangover* gene was found as required for normal development of ethanol tolerance in the fruit flies [99]. Human *HANGOVER/ZNF699* gene polymorphisms were associated in a large population of Irish alcohol dependant patients compared to matched controls [100]. The genetic studies in alcohol dependence also recently took advantage of novel technology for high-throughput genotyping based on microarrays. Thus, association genome scanning, based on the simultaneous genotyping of over 100,000 SNPs, was carried out on the COGA [101]. Convergent loci and associations, as previously reported, were found. Furthermore, novel loci were identified confirming the high definition of this new tool in screening capacity [101]. Recently, rare structural variants, named copy number variants (CNVs), which correspond to micro-duplications or microdeletions of 100 kb at least leading to duplicate or disrupt genes, were identified by comparative genomic hybridization in several psychiatric disorders [102, 103]. Thus, search for CNVs should be one of the next future directions in the molecular genetic of alcohol dependence.

Genomic functional, including gene and protein expression profiling studies, on cell lines, animal models and human post-mortem samples confirmed that several biological pathways are involved in the vulnerability to alcohol preference, and opened avenues for patient-tailored treatment, in both pharmacogenomic and clinical proteomic. This genomic functional approach has to be combined with genetic analyses, and have to be performed on large number of patients, assessing a large set of clinical features and phenotypic information, also including endophenotypes to narrow the associated gene, or biomarker, to a specific step in the pathway to alcohol dependence. Finally, pharmacogenomics is the next issue in the comprehension of the molecular biology of alcohol dependence.

## References

[1] Kessler RC, Berglund P, Demler O, Jin R, Merikangas KR, Walters EE (2005). Lifetime prevalence and age-of-onset distributions of DSM-IV disorders in the National Comorbidity Survey Replication. Arch. Gen. Psychiatry.

[2] Hiroi N, Agatsuma S (2005). Genetic susceptibility to substance dependence. Mol. Psychiatry.

[3] Goldman D, Oroszi G, Ducci F (2005). The genetics of addictions: uncovering the genes. Nat. Rev. Genet.

[4] Ramoz N, Schumann G, Gorwood P (2006). Genetic and pharmacogenetic aspects of alcohol-dependence. Curr. Pharmacogenom.

[5] Gorwood P, Wohl M, Le Strat Y, Rouillon F (2007). Gene-environment interactions in addictive disorders: epidemiological and methodological aspects. C. R. Biol.

[6] Dick DM, Bierut LJ (2006). The genetics of alcohol dependence. Curr. Psychiatry Rep.

[7] American Psychiatric Association (1987). Diagnostic and Statistical Manual of Mental Disorders.

[8] Feighner JP, Robins E, Guze SB, Woodruff RA Jr, Winokur G, Munoz R (1972). Diagnostic criteria for use in psychiatric research. Arch. Gen. Psychiatry.

[9] Reich T, Edenberg HJ, Goate A, Williams JT, Rice JP, Van Eerdewegh P, Foroud T, Hesselbrock V, Schuckit MA, Bucholz K, Porjesz B, Li TK, Conneally PM, Nurnberger JI Jr, Tischfield JA, Crowe RR, Cloninger CR, Wu W, Shears S, Carr K, Crose C, Willig C, Begleiter H (1998). Genome-wide search for genes affecting the risk for alcohol dependence. Am. J. Med. Genet. B Neuropsychiatr. Genet.

[10] Zinn-Justin A, Abel L (1999). Genome search for alcohol dependence using the weighted pairwise correlation linkage method: interesting findings on chromosome 4. Genet. Epidemiol.

[11] Dunn G, Hinrichs AL, Bertelsen S, Jin CH, Kauwe JS, Suarez BK, Bierut LJ (2005). Microsatellites versus single-nucleotide polymorphisms in linkage analysis for quantitative and qualitative measures. BMC Genet.

[12] Dick DM, Aliev F, Wang JC, Saccone S, Hinrichs A, Bertelsen S, Budde J, Saccone N, Foroud T, Nurnberger J Jr, Xuei X, Conneally PM, Schuckit M, Almasy L, Crowe R, Kuperman S, Kramer J, Tischfield JA, Hesselbrock V, Edenberg HJ, Porjesz B, Rice JP, Bierut L, Goate A (2008). A Systematic Single Nucleotide Polymorphism Screen to Fine-Map Alcohol Dependence Genes on Chromosome 7 Identifies Association With a Novel Susceptibility Gene ACN9. Biol. Psychiatry.

[13] Ittiwut C, Listman J, Mutirangura A, Malison R, Covault J, Kranzler HR, Sughondhabirom A, Thavichachart N, Gelernter J (2008). Interpopulation linkage disequilibrium patterns of GABRA2 and GABRG1 genes at the GABA cluster locus on human chromosome 4. Genomics.

[14] Hinrichs AL, Wang JC, Bufe B, Kwon JM, Budde J, Allen R, Bertelsen S, Evans W, Dick D, Rice J, Foroud T, Nurnberger J, Tischfield JA, Kuperman S, Crowe R, Hesselbrock V, Schuckit M, Almasy L, Porjesz B, Edenberg HJ, Begleiter H, Meyerhof W, Bierut LJ, Goate AM (2006). Functional variant in a bitter-taste receptor (hTAS2R16) influences risk of alcohol dependence. Am. J. Hum. Genet.

[15] Blednov YA, Walker D, Martinez M, Levine M, Damak S, Margolskee RF (2008). Perception of sweet taste is important for voluntary alcohol consumption in mice. Genes Brain Behav.

[16] Wang JC, Hinrichs AL, Stock H, Budde J, Allen R, Bertelsen S, Kwon JM, Wu W, Dick DM, Rice J, Jones K, Nurnberger JI Jr, Tischfield J, Porjesz B, Edenberg HJ, Hesselbrock V, Crowe R, Schuckit M, Begleiter H, Reich T, Goate AM, Bierut LJ (2004). Evidence of common and specific genetic effects: association of the muscarinic acetylcholine receptor M2 (CHRM2) gene with alcohol dependence and major depressive syndrome. Hum. Mol. Genet.

[17] Luo X, Kranzler HR, Zuo L, Zhang H, Wang S, Gelernter J (2007). CHRM2 variation predisposes to personality traits of agreeableness and conscientiousness. Hum. Mol. Genet.

[18] Gosso FM, de Geus EJ, Polderman TJ, Boomsma DI, Posthuma D, Heutink P (2007). Exploring the functional role of the CHRM2 gene in human cognition: results from a dense genotyping and brain expression study. BMC Med. Genet.

[19] Dick DM, Aliev F, Kramer J, Wang JC, Hinrichs A, Bertelsen S, Kuperman S, Schuckit M, Nurnberger JI Jr, Edenberg HJ, Porjesz B, Begleiter H, Hesselbrock V, Goate A, Bierut L (2007). Association of CHRM2 with IQ: converging evidence for a gene influencing intelligence. Behav. Genet.

[20] Jones KA, Porjesz B, Almasy L, Bierut L, Goate A, Wang JC, Dick DM, Hinrichs A, Kwon J, Rice JP, Rohrbaugh J, Stock H, Wu W, Bauer LO, Chorlian DB, Crowe RR, Edenberg HJ, Foroud T, Hesselbrock V, Kuperman S, Nurnberger J Jr, O'Connor SJ, Schuckit MA, Stimus AT, Tischfield JA, Reich T, Begleiter H (2004). Linkage and linkage disequilibrium of evoked EEG oscillations with CHRM2 receptor gene polymorphisms: implications for human brain dynamics and cognition. Int. J. Psychophysiol.

[21] Agrawal A, Hinrichs AL, Dunn G, Bertelsen S, Dick DM, Saccone SF, Saccone NL, Grucza RA, Wang JC, Cloninger CR, Edenberg HJ, Foroud T, Hesselbrock V, Kramer J, Bucholz KK, Kuperman S, Nurnberger JI Jr, Porjesz B, Schuckit MA, Goate AM, Bierut LJ (2008). Linkage scan for quantitative traits identifies new regions of interest for substance dependence in the Collaborative Study on the Genetics of Alcoholism (COGA) sample. Drug Alcohol Depend.

[22] Dawn Teare M, Barrett JH (2005). Genetic linkage studies. Lancet.

[23] Lin Z, Walther D, Yu XY, Drgon T, Uhl GR (2004). The human serotonin receptor 2B: coding region polymorphisms and association with vulnerability to illegal drug abuse. Pharmacogenetics.

[24] Lappalainen J, Krupitsky E, Kranzler HR, Luo X, Remizov M, Pchelina S, Taraskina A, Zvartau E, Räsanen P, Makikyro T, Somberg LK, Krystal JH, Stein MB, Gelernter J (2007). Mutation screen of the GAD2 gene and association study of alcoholism in three populations. Am. J. Med. Genet. B Neuropsychiatr. Genet.

[25] Compton WM, Thomas YF, Stinson FS, Grant BF (2007). Prevalence, correlates, disability, and comorbidity of DSM-IV drug abuse and dependence in the United States: results from the national epidemiologic survey on alcohol and related conditions. Arch. Gen. Psychiatry.

[26] Ehlers CL, Gilder DA, Wall TL, Phillips E, Feiler H, Wilhelmsen KC (2004). Genomic screen for loci associated with alcohol dependence in Mission Indians. Am. J. Med. Genet. B Neuropsychiatr. Genet.

[27] Radel M, Vallejo RL, Iwata N, Aragon R, Long JC, Virkkunen M, Goldman D (2005). Haplotype-based localization of an alcohol dependence gene to the 5q34 {gamma}-aminobutyric acid type A gene cluster. Arch. Gen. Psychiatry.

[28] Saccone NL, Kwon JM, Corbett J, Goate A, Rochberg N, Edenberg HJ, Foroud T, Li TK, Begleiter H, Reich T, Rice JP (2000). A genome screen of maximum number of drinks as an alcoholism phenotype. Am. J. Med. Genet.

[29] Ehrig T, Bosron WF, Li TK (1990). Alcohol and aldehyde dehydrogenase. Alcohol Alcohol.

[30] Edenberg HJ, Xuei X, Chen HJ, Tian H, Wetherill LF, Dick DM, Almasy L, Bierut L, Bucholz KK, Goate A, Hesselbrock V, Kuperman S, Nurnberger J, Porjesz B, Rice J, Schuckit M, Tischfield J, Begleiter H, Foroud T (2006). Association of alcohol dehydrogenase genes with alcohol dependence: a comprehensive analysis. Hum. Mol. Genet.

[31] Cheng AT, Gau SF, Chen TH, Chang JC, Chang YT (2004). A 4-year longitudinal study on risk factors for alcoholism. Arch. Gen. Psychiatry.

[32] Spivak B, Frisch A, Maman Z, Aharonovich E, Alderson D, Carr LG, Weizman A, Hasin D (2007). Effect of ADH1B genotype on alcohol consumption in young Israeli Jews. Alcohol. Clin. Exp. Res.

[33] Cichoz-Lach H, Partycka J, Nesina I, Celinski K, Slomka M, Wojcierowski J (2007). Alcohol dehydrogenase and aldehyde dehydrogenase gene polymorphism in alcohol liver cirrhosis and alcohol chronic pancreatitis among Polish individuals. Scand. J. Gastroenterol.

[34] Osier M, Pakstis AJ, Kidd JR, Lee JF, Yin SJ, Ko HC, Edenberg HJ, Lu RB, Kidd KK (1999). Linkage disequilibrium at the ADH2 and ADH3 loci and risk of alcoholism. Am.J. Hum. Genet.

[35] Birley AJ, Whitfield JB, Neale MC, Duffy DL, Heath AC, Boomsma DI, Martin NG (2005). Genetic time-series analysis identifies a major QTL for *in vivo* alcohol metabolism not predicted by *in vitro* studies of structural protein polymorphism at the ADH1B or ADH1C loci. Behav. Genet.

[36] Luo X, Kranzler HR, Zuo L, Yang BZ, Lappalainen J, Gelernter J (2005). ADH4 gene variation is associated with alcohol and drug dependence: results from family controlled and population-structured association studies. Pharmacogenet. Genomics.

[37] Luo X, Kranzler HR, Zuo L, Wang S, Gelernter J (2007). Personality traits of agreeableness and extraversion are associated with ADH4 variation. Biol. Psychiatry.

[38] Birley AJ, James MR, Dickson PA, Montgomery GW, Heath AC, Whitfield JB, Martin NG (2008). Association of the gastric alcohol dehydrogenase gene ADH7 with variation in alcohol metabolism. Hum. Mol. Genet.

[39] Luo X, Kranzler HR, Zuo L, Wang S, Schork NJ, Gelernter J (2006). Diplotype trend regression analysis of the ADH gene cluster and the ALDH2 gene: multiple significant associations with alcohol dependence. Am. J. Hum. Genet.

[40] Luo X, Kranzler HR, Zuo L, Wang S, Schork NJ, Gelernter J (2007). Multiple ADH genes modulate risk for drug dependence in both African- and European-Americans. Hum. Mol. Genet.

[41] Luo X, Kranzler HR, Zuo L, Zhang H, Wang S, Gelernter J (2008). ADH7 variation modulates extraversion and conscientiousness in substance-dependent subjects. Am. J. Med. Genet. B Neuropsychiatr. Genet.

[42] Takeshita T, Morimoto K, Mao X, Hashimoto T, Furuyama J (1994). Characterization of the three genotypes of low Km aldehyde dehydrogenase in a Japanese population. Hum. Genet.

[43] Chen WJ, Loh EW, Hsu YP, Cheng AT (1997). Alcohol dehydrogenase and aldehyde dehydrogenase genotypes and alcoholism among Taiwanese aborigines. Biol. Psychiatry.

[44] Chen CC, Lu RB, Chen YC, Wang MF, Chang YC, Li TK, Yin SJ (1999). Interaction between the functional polymorphisms of the alcohol-metabolism genes in protection against alcoholism. Am. J. Hum. Genet.

[45] Martinez D, Gil R, Slifstein M, Hwang DR, Huang Y, Perez A, Kegeles L, Talbot P, Evans S, Krystal J, Laruelle M, Abi-Dargham A (2005). Alcohol dependence is associated with blunted dopamine transmission in the ventral striatum. Biol. Psychiatry.

[46] Gorwood P, Batel P, Gouya L, Courtois F, Feingold J, Ades J (2000). Reappraisal of the association between the DRD2 gene, alcoholism and addiction. Eur. Psychiatry.

[47] Munafò MR, Matheson IJ, Flint J (2007). Association of the DRD2 gene Taq1A polymorphism and alcoholism: a meta-analysis of case-control studies and evidence of publication bias. Mol. Psychiatry.

[48] Klein TA, Neumann J, Reuter M, Hennig J, von Cramon DY, Ullsperger M (2007). Genetically determined differences in learning from errors. Science.

[49] Yang BZ, Kranzler HR, Zhao H, Gruen JR, Luo X, Gelernter J (2007). Association of haplotypic variants in DRD2, ANKK1, TTC12 and NCAM1 to alcohol dependence in independent case control and family samples. Hum. Mol. Genet.

[50] Dick DM, Wang JC, Plunkett J, Aliev F, Hinrichs A, Bertelsen S, Budde JP, Goldstein EL, Kaplan D, Edenberg HJ, Nurnberger J Jr, Hesselbrock V, Schuckit M, Kuperman S, Tischfield J, Porjesz B, Begleiter H, Bierut LJ, Goate A (2007). Family-based association analyses of alcohol dependence phenotypes across DRD2 and neighboring gene ANKK1 Alcohol. Clin. Exp. Res.

[51] Dubertret C, Gouya L, Hanoun N, Deybach JC, Adès J, Hamon M, Gorwood P (2004). The 3' region of the DRD2 gene is involved in genetic susceptibility to schizophrenia. Schizophr. Res.

[52] Batel P, Houchi H, Daoust M, Ramoz N, Naassila M, Gorwood P (2008). A haplotype of the DRD1 gene is associated with alcohol dependence. Alcohol. Clin. Exp. Res.

[53] Kim DJ, Park BL, Yoon S, Lee HK, Joe KH, Cheon YH, Gwon DH, Cho SN, Lee HW, NamGung S, Shin HD (2007). 5' UTR polymorphism of dopamine receptor D1 (DRD1) associated with severity and temperament of alcoholism. Biochem. Biophys. Res. Commun.

[54] Vengeliene V, Leonardi-Essmann F, Perreau-Lenz S, Gebicke-Haerter P, Drescher K, Gross G, Spanagel R (2006). The dopamine D3 receptor plays an essential role in alcohol-seeking and relapse. FASEB J.

[55] Gorwood P, Limosin F, Batel P, Duaux E, Gouya L, Ades J (2001). The genetics of addiction: alcohol-dependence and D3 dopamine receptor gene. Pathol. Biol. (Paris).

[56] van den Wildenberg E, Janssen RG, Hutchison KE, van Breukelen GJ, Wiers RW (2007). Polymorphisms of the dopamine D4 receptor gene (DRD4 VNTR) and cannabinoid CB1 receptor gene (CNR1) are not strongly related to cue-reactivity after alcohol exposure. Addict. Biol.

[57] Wiesbeck GA, Dürsteler-MacFarland KM, Wurst FM, Walter M, Petitjean S, Müller S, Wodarz N, Böning J (2006). No association of dopamine receptor sensitivity *in vivo* with genetic predisposition for alcoholism and DRD2/DRD3 gene polymorphisms in alcohol dependence. Addict. Biol.

[58] Kohnke MD, Batra A, Kolb W, Kohnke AM, Lutz U, Schick S, Gaertner I (2005). Association of the dopamine transporter gene with alcoholism. Alcohol.

[59] Muramatsu T, Higuchi S (1995). Dopamine transporter gene polymorphism and alcoholism. Biochem. Biophys. Res. Commun.

[60] Chen WJ, Chen CH, Huang J, Hsu YP, Seow SV, Chen CC, Cheng AT (2001). Genetic polymorphisms of the promoter region of dopamine D2 receptor and dopamine transporter genes and alcoholism among four aboriginal groups and Han Chinese in Taiwan. Psychiatr. Genet.

[61] Foley PF, Loh EW, Innes DJ, Williams SM, Tannenberg AE, Harper CG, Dodd PR (2004). Association studies of neurotransmitter gene polymorphisms in alcoholic Caucasians. Ann. N. Y. Acad. Sci.

[62] Schmidt LG, Harms H, Kuhn S, Rommelspacher H, Sander T (1998). Modification of alcohol withdrawal by the A9 allele of the dopamine transporter gene. Am. J. Psychiatry.

[63] Gorwood P, Limosin F, Batel P, Hamon M, Ades J, Boni C (2003). The A9 allele of the dopamine transporter gene is associated with delirium tremens and alcohol-withdrawal seizure. Biol. Psychiatry.

[64] Le Strat Y, Ramoz N, Pickering P, Burger V, Boni C, Aubin HJ, Ades J, Batel P, Gorwood P (2008). The 3' part of the dopamine transporter gene DAT1/SLC6A3 is associated with withdrawal seizures in patients with alcohol dependence. Alcohol. Clin. Exp. Res.

[65] Lovinger DM, Homanics GE (2007). Tonic for what ails us? high-affinity GABAA receptors and alcohol. Alcohol.

[66] Buck KJ, Hood HM (1998). Genetic association of a GABA(A) receptor gamma2 subunit variant with severity of acute physiological dependence on alcohol. Mamm. Genome.

[67] Loh EW, Smith II, Murray R, McLaughlin M, McNulty S, Ball D (1999). Association between variants at the GABAAbeta2, GABAAalpha6 and GABAAgamma2 gene cluster and alcohol dependence in a scottish population. Mol. Psychiatry.

[68] Sander T, Ball D, Murray R, Patel J, Samochowiec J, Winterer G, Rommelspacher H, Schmidt LG, Loh EW (1999). Association analysis of sequence variants of GABA(A) alpha6, beta2, and gamma2 gene cluster and alcohol dependence. Alcohol. Clin. Exp. Res.

[69] Edenberg HJ, Dick DM, Xuei X, Tian H, Almasy L, Bauer LO, Crowe RR, Goate A, Hesselbrock V, Jones K, Kwon J, Li TK, Nurnberger JI Jr, O'Connor SJ, Reich T, Rice J, Schuckit MA, Porjesz B, Foroud T, Begleiter H (2004). Variations in GABRA2, encoding the alpha 2 subunit of the GABA(A) receptor, are associated with alcohol dependence and with brain oscillations. Am. J. Hum. Genet.

[70] Agrawal A, Edenberg HJ, Foroud T, Bierut LJ, Dunne G, Hinrichs AL, Nurnberger JI, Crowe R, Kuperman S, Schuckit MA, Begleiter H, Porjesz B, Dick DM (2006). Association of GABRA2 with drug dependence in the collaborative study of the genetics of alcoholism sample. Behav. Genet.

[71] Soyka M, Preuss UW, Hesselbrock V, Zill P, Koller G, Bondy B (2008). GABA-A2 receptor subunit gene (GABRA2) polymorphisms and risk for alcohol dependence. J. Psychiatr. Res.

[72] Mulligan MK, Ponomarev I, Hitzemann RJ, Belknap JK, Tabakoff B, Harris RA, Crabbe JC, Blednov YA, Grahame NJ, Phillips TJ, Finn DA, Hoffman PL, Iyer VR, Koob GF, Bergeson SE (2006). Toward understanding the genetics of alcohol drinking through transcriptome meta-analysis. Proc. Natl. Acad. Sci. U. S. A.

[73] Bowers BJ, Radcliffe RA, Smith AM, Miyamoto-Ditmon J, Wehner JM (2006). Microarray analysis identifies cerebellar genes sensitive to chronic ethanol treatment in PKCgamma mice. Alcohol.

[74] Singh SM, Treadwell J, Kleiber ML, Harrison M, Uddin RK (2007). Analysis of behavior using genetical genomics in mice as a model: from alcohol preferences to gene expression differences. Genome.

[75] Sommer W, Hyytiä P, Kiianmaa K (2006). The alcohol-preferring AA and alcohol-avoiding ANA rats: neurobiology of the regulation of alcohol drinking. Addict. Biol.

[76] Rodd ZA, Bertsch BA, Strother WN, Le-Niculescu H, Balaraman Y, Hayden E, Jerome RE, Lumeng L, Nurnberger JI Jr, Edenberg HJ, McBride WJ, Niculescu AB (2007). Candidate genes, pathways and mechanisms for alcoholism: an expanded convergent functional genomics approach. Pharmacogenomics J.

[77] Worst TJ, Vrana KE (2005). Alcohol and gene expression in the central nervous system. Alcohol Alcohol.

[78] Fan L, Jaquet V, Dodd PR, Chen W, Wilce PA (2001). Molecular cloning and characterization of hNP22: a gene up-regulated in human alcoholic brain. J. Neurochem.

[79] Sokolov BP, Jiang L, Trivedi NS, Aston C (2003). Transcription profiling reveals mitochondrial, ubiquitin and signaling systems abnormalities in postmortem brains from subjects with a history of alcohol abuse or dependence. J. Neurosci. Res.

[80] Flatscher-Bader T, van der Brug M, Hwang JW, Gochee PA, Matsumoto I, Niwa S, Wilce PA (2005). Alcohol-responsive genes in the frontal cortex and nucleus accumbens of human alcoholics. J. Neurochem.

[81] Liu J, Lewohl JM, Dodd PR, Randall PK, Harris RA, Mayfield RD (2004). Gene expression profiling of individual cases reveals consistent transcriptional changes in alcoholic human brain. J. Neurochem.

[82] Liu J, Lewohl JM, Harris RA, Iyer VR, Dodd PR, Randall PK, Mayfield RD (2006). Patterns of gene expression in the frontal cortex discriminate alcoholic from nonalcoholic individuals. Neuropsychopharmacology.

[83] Neuhold LA, Guo QM, Alper J, Velazquez JM (2004). High-throughput proteomics for alcohol research. Alcohol Clin. Exp. Res.

[84] Witzmann FA, Strother WN (2004). Proteomics and alcoholism. Int. Rev. Neurobiol.

[85] Klouckova I, Hrncirova P, Mechref Y, Arnold RJ, Li TK, McBride WJ, Novotny MV (2006). Changes in liver protein abundance in inbred alcohol-preferring rats due to chronic alcohol exposure, as measured through a proteomics approach. Proteomics.

[86] Freeman WM, Gooch RS, Lull ME, Worst TJ, Walker SJ, Xu AS, Green H, Pierre PJ, Grant KA, Vrana KE (2006). Apo-AII is an elevated biomarker of chronic non-human primate ethanol self-administration. Alcohol Alcohol.

[87] Nomura F, Tomonaga T, Sogawa K, Ohashi T, Nezu M, Sunaga M, Kondo N, Iyo M, Shimada H, Ochiai T (2004). Identification of novel and downregulated biomarkers for alcoholism by surface enhanced laser desorption/ionization-mass spectrometry. Proteomics.

[88] Alexander-Kaufman K, James G, Sheedy D, Harper C, Matsumoto I (2006). Differential protein expression in the prefrontal white matter of human alcoholics: a proteomics study. Mol. Psychiatry.

[89] Gottesman II, Gould TD (2003). The endophenotype concept in psychiatry: etymology and strategic intentions. Am. J. Psychiatry.

[90] Chorlian DB, Tang Y, Rangaswamy M, O'Connor S, Rohrbaugh J, Taylor R, Porjesz B (2007). Heritability of EEG coherence in a large sib-pair population. Biol. Psychol.

[91] Anokhin A, Steinlein O, Fischer C, Mao Y, Vogt P, Schalt E, Vogel F (1992). A genetic study of the human low-voltage electroencephalogram. Hum. Genet.

[92] Ehlers CL, Phillips E, Schuckit MA (2004). EEG alpha variants and alpha power in Hispanic American and white non-Hispanic American young adults with a family history of alcohol dependence. Alcohol.

[93] Rangaswamy M, Porjesz B, Chorlian DB, Wang K, Jones KA, Kuperman S, Rohrbaugh J, O'Connor SJ, Bauer LO, Reich T, Begleiter H (2004). Resting EEG in offspring of male alcoholics: beta frequencies. Int. J. Psychophysiol.

[94] Yu Y, Meng Y, Ma Q, Farrell J, Farrer LA, Wilcox MA (2005). Whole-genome variance components linkage analysis using single-nucleotide polymorphisms versus microsatellites on quantitative traits of derived phenotypes from factor analysis of electroencephalogram waves. BMC Genet.

[95] Dick DM, Jones K, Saccone N, Hinrichs A, Wang JC, Goate A, Bierut L, Almasy L, Schuckit M, Hesselbrock V, Tischfield J, Foroud T, Edenberg H, Porjesz B, Begleiter H (2006). Endophenotypes successfully lead to gene identification: results from the collaborative study on the genetics of alcoholism. Behav. Genet.

[96] van Beijsterveldt CE, van Baal GC, Molenaar PC, Boomsma DI, de Geus EJ (2001). Stability of genetic and environmental influences on P300 amplitude: a longitudinal study in adolescent twins. Behav. Genet.

[97] Begleiter H, Porjesz B, Bihari B, Kissin B (1984). Event-related brain potentials in boys at risk for alcoholism. Science.

[98] Chen AC, Porjesz B, Rangaswamy M, Kamarajan C, Tang Y, Jones KA, Chorlian DB, Stimus AT, Begleiter H (2007). Reduced frontal lobe activity in subjects with high impulsivity and alcoholism. Alcohol. Clin. Exp. Res.

[99] Scholz H, Franz M, Heberlein U (2005). The hangover gene defines a stress pathway required for ethanol tolerance development. Nature.

[100] Riley BP, Kalsi G, Kuo PH, Vladimirov V, Thiselton DL, Vittum J, Wormley B, Grotewiel MS, Patterson DG, Sullivan PF, van den Oord E, Walsh D, Kendler KS, Prescott CA (2006). Alcohol dependence is associated with the ZNF699 gene, a human locus related toDrosophila hangover, in the Irish Affected Sib Pair Study of Alcohol Dependence(IASPSAD) sample. Mol. Psychiatry.

[101] Johnson C, Drgon T, Liu QR, Walther D, Edenberg H, Rice J, Foroud T, Uhl GR (2006). Pooled association genome scanning for alcohol dependence using 104,268 SNPs: validation and use to identify alcoholism vulnerability loci in unrelated individuals from the collaborative study on the genetics of alcoholism. Am. J. Med. Genet. B Neuropsychiatr. Genet.

[102] Sebat J (2007). Major changes in our DNA lead to major changes in our thinking. Nat. Genet.

[103] Cantor RM, Geschwind DH (2008). Schizophrenia: genome, interrupted. Neuron.

